# Genetic Engineering of Mesenchymal Stem Cells to Induce Their Migration and Survival

**DOI:** 10.1155/2016/4956063

**Published:** 2016-05-03

**Authors:** Adam Nowakowski, Piotr Walczak, Barbara Lukomska, Miroslaw Janowski

**Affiliations:** ^1^NeuroRepair Department, Mossakowski Medical Research Centre, Polish Academy of Sciences, Warsaw, Poland; ^2^Russell H. Morgan Department of Radiology and Radiological Science, Division of MR Research, The Johns Hopkins University School of Medicine, 733 N. Broadway Street, Room 649, Baltimore, MD 21205, USA; ^3^Cellular Imaging Section and Vascular Biology Program, Institute for Cell Engineering, The Johns Hopkins University School of Medicine, Baltimore, MD, USA

## Abstract

Mesenchymal stem cells (MSCs) are very attractive for regenerative medicine due to their relatively easy derivation and broad range of differentiation capabilities, either naturally or induced through cell engineering. However, efficient methods of delivery to diseased tissues and the long-term survival of grafted cells still need improvement. Here, we review genetic engineering approaches designed to enhance the migratory capacities of MSCs, as well as extend their survival after transplantation by the modulation of prosurvival approaches, including prevention of senescence and apoptosis. We highlight some of the latest examples that explore these pivotal points, which have great relevance in cell-based therapies.

## 1. Introduction

Interest in stem cell-based regenerative medicine is growing. Furthermore, implementation of genetic engineering methods is capable of further enhancing the therapeutic potential of stem cells [1]. Mesenchymal stem cells (MSCs) are very promising because they are easy to isolate and they have a broad range of differentiation capabilities, either naturally or through cell engineering [2]. However, when considering the use of MSCs in therapy, many practical problems should be resolved, among which is proper and efficient delivery and keeping the cells alive at the sites of action. Under hypoxic conditions, endogenous MSCs have an increased ability to migrate and influence the factors secreted from the damaged tissue. As a response to the reduced partial pressure of oxygen in tissues surrounding the injured area, MSCs change their membrane receptors and are capable of migration toward the site of the damage [3]. This occurs, however, exclusively within the damaged and surrounding regions; thus, the migration refers only to MSCs that are relatively close to the site(s) of injury and can be effectively activated. Therefore, the number of recruited cells is limited. For this reason, it was proposed that the therapeutic effect could be enhanced by the administration of exogenous MSCs to the sites of injury, and this was, indeed, confirmed [4].

**Figure 1:**
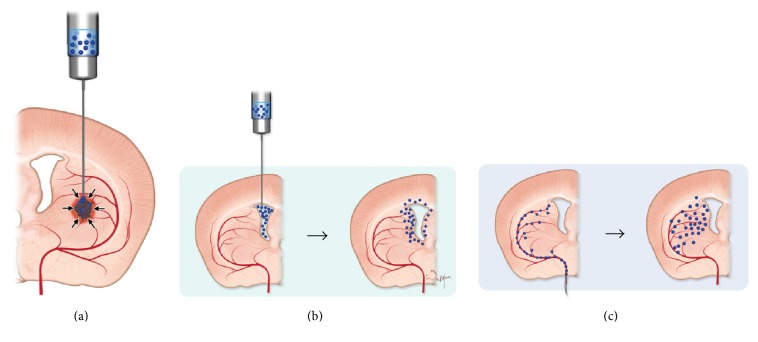
The role of migration in various routes of cell delivery. (a) Intraparenchymal injection triggers inflammatory responses and may augment the host reaction against the graft (arrows). Single stem cell infiltration of brain parenchyma after intraventricular (b) and intra-arterial (c) infusion.

To date, local injection of MSCs is the most prevalent cell delivery method, but local injection has many drawbacks. In particular, the deposition of a bulk cell suspension in very delicate organs, such as the brain, tears the complex tissue structure, causes pressure on local structures, and frequently results in microbleeding, which triggers inflammatory responses and may augment the host reaction against the graft (Figure 1(a)) [5]. In addition, the needle insertion into acutely damaged brain tissue, as in the case of stroke or traumatic brain injury, is life-threatening due to the risk of hematoma formation. Thus, substantial effort has been devoted to avoiding such stressful conditions which can be detrimental to both graft and host. An attractive alternative to intraparenchymal injection is deposition of cells in fluid compartments, from which they could migrate toward injured/diseased areas without causing any strain on the intact tissue (Figure 1(b)). There are multiple studies with transplantation of cells into cerebrospinal fluid spaces [6, 7] but inadequate intraparenchymal migration limited the therapeutic effect [8].

Intravascular injection is another way to use body fluids for efficient delivery of cells to large body areas, including the brain. The intravenous route is noninvasive, but wide, whole-body cell distribution might limit the amount of cells that reach the diseased area. The intra-arterial route is especially interesting as it is still minimally invasive but allows targeting of specific body areas, including particular brain regions, and in that case the cells are expected to extravasate and disperse within a brain parenchyma (Figure 1(c)). Moreover, the safety issues have been recently extensively investigated and addressed [9, 10].

However, MSCs are not equipped to allow for efficient migration from cerebrospinal fluid or the blood to the brain parenchyma. While the precision of intraparenchymal injection can be very high, due to the fragility of damaged tissue, cells must be deposited at some distance from the disease site to avoid further injury. Implanted cells are then required to migrate a certain distance to reach the diseased tissue. Despite the fact that native MSCs showed some migration toward injured areas after administration [11], there is a need to further increase that migration ability after engraftment of exogenous MSCs, and that can be achieved by genetic cell engineering [12].

However, an important aspect of MSC-based therapies is the maintenance of their proliferative and differentiation capacities. Prolonged culture of MSCs results in an inevitable senescence, consequently leading to the loss of their proliferative activity [13]. To address this issue, several efforts have been made to increase the expression of stemness-related genes in such a way that the beneficial properties of MSCs are maintained and even increased by extending their* in vitro* expansion potential.

Furthermore, it is important to note that, in the majority of cases, the delivered therapeutic cells encounter adverse conditions after transplantation into injured target tissues with a hostile microenvironment. High levels of oxidative stress, local hypoxia, and proapoptotic cytokines all contribute to the elimination of the transplanted therapeutic cells, which, in turn, limits their therapeutic activity. Thus, prosurvival approaches are needed to prolong the engraftment time of exogenous cells.

## 2. MSCs Engineered to Increase Migratory Properties

**Figure 2:**
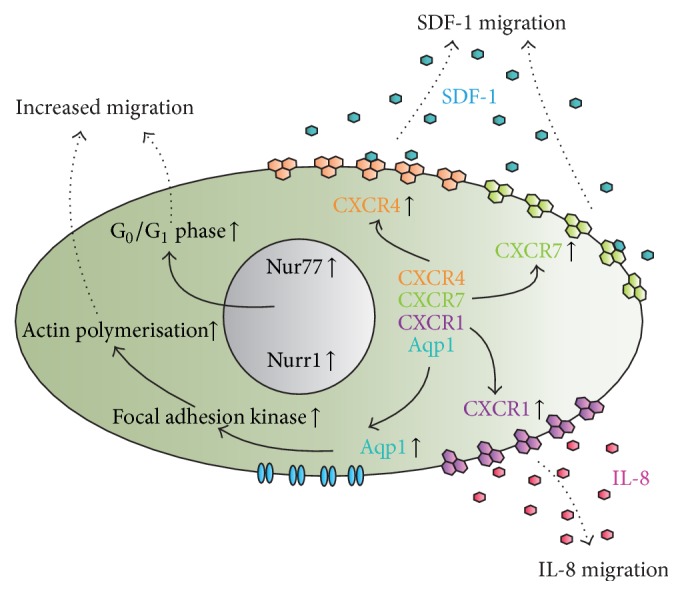
MSCs engineered to enhance migratory properties. Increased MSC migration could be accomplished by membrane-bound receptor engineering (i.e., CXCR1, CXCR4, and CXCR7), water channel receptors (Aqp1), or the upregulation of defined nuclear receptors (i.e., Nur77 and Nurr1).

SDF-1 is one of the most potent chemokines involved in the process of cell migration [14]. Under physiological conditions, SDF-1 is produced within the damaged tissue and is released from the injured area, exerting chemoattractive signals for the cells that express the CXCR4 receptor on their outer cell membrane [15, 16]. CXCR4 basal protein presence in outer MSC membranes differs between various MSCs. There are some data that unmodified bone marrow-derived mouse [17] and human [18] MSCs do not possess CXCR4 in their outer membranes or present low levels, that is, rat MSCs [16, 19, 20] and human adipose-derived MSCs [15]. However, other studies bring contrary observations for rat MSCs [21, 22]. Moreover, CXCR4 presence is altered during* in vitro* culture [15]. MSCs could heavily increase the presence of CXCR4 particularly when exposed to a low concentration of oxygen [20, 22] or after adequate stimulation to elicit the endogenous CXCR4 gene overexpression [15, 23]. Despite this, there have been numerous reports of engineering MSCs to increase the expression of the CXCR4 gene, which have resulted in a higher density of the CXCR4 receptor and effectively increased the migration of MSCs toward SDF-1 [24–26] (Figure 2).

The beneficial immunoregulatory effects of CXCR4-expressing MSCs were observed in a study on kidney transplantation [27]. In a different study, CXCR4-engineered MSCs had a positive impact on early liver regeneration, which was attributed to their enhanced homing to liver grafts, with an emphasis on the contribution to the improvement of hepatocyte proliferation [28]. Another example of the beneficial effects of CXCR4-overexpressing MSCs was enhanced tissue repair in an acute kidney injury model [29]. In this case, CXCR4-MSCs homed to the lesion site with enhanced affinity, compared to the control MSCs, exhibiting beneficial paracrine actions. Another example is the data on the use of CXCR4-MSCs in the healing of skin wounds. As in the above cases, it was shown that CXCR4-engineered MSCs migrated with higher affinity to the sites of injuries, accelerating the process of wound healing [30]. In the case of a rat cerebral ischemia model, the delivered CXCR4-MSCs possessed higher mobilization and enhanced neuroprotection compared to the control cells [31]. In addition to the CXCR4 element from the SDF-1-CXCR4 signal axis, MSCs were also engineered to overexpress SDF-1. Nakamura et al. presented the results of SDF-1-overexpressing MSCs that had enhanced migration properties in* in vitro* migration assays, and SDF-1-MSCs were used in the* in vivo* experiments for wound healing. It was observed that SDF-1-MSCs contributed to a significant wound size decrease, which raised expectations that modified MSCs could be used in the treatment of skin injuries [32].

In addition to the CXCR4 binding abilities of SDF-1, CXC chemokine receptor 7 (CXCR7) was observed to bind SDF-1 as well [33], so that the SDF-1/CXCR7 signaling axis was used to engineer the MSCs. Wang et al. used CXCR7-overexpressing MSCs in a cerebral ischemia-reperfusion rat hippocampus model. It was proven that the overexpressed CXCR7 receptor promoted the migration of MSCs toward an SDF-1 gradient, acting jointly with the SDF-1/CXCR4 signaling axis [34]. Overexpression of the CXCR7 receptor in MSCs resulted in their enhanced migration toward the secondary lymphoid organs. CXCR7-engineered MSCs homed extensively to these organs, potentially inhibiting the immune system response in graft-versus-host disease and thus decreasing clinical symptoms [35].

Another CXC chemokine receptor selected to enhance the migratory properties of MSCs was the CXC chemokine receptor 1 (CXCR1). The CXCR1 is a receptor for IL-8, which, in turn, was shown to be expressed and released in gliomas [36]. This phenomenon was used to improve targeting of MSC toward gliomas [37]. In a different study, CXCR1-MSCs were shown to accumulate in the infarcted myocardium with high affinity, where the survival and engraftment of exogenously delivered CXCR1-MSCs were elevated, providing a putative new strategy for the injured myocardium [38].

The migratory properties of MSCs were also manipulated via modification of the aquaporin-1 (Aqp1) gene. Overexpression of Aqp1 resulted in an increase of the migration capabilities of Aqp1-MSCs toward the sites of injury [39]. Aqp1 is a water channel molecule that transports water across the cell membrane. It was shown that Aqp1 interacted with *β*-catenin, which was an important regulator of cell migration [40].

Two nuclear receptors, Nur77 and Nurr1, were also brought into play to improve the migratory capabilities of MSCs [41]. The high expression of Nur77 and Nurr1 was characteristic of the cells with enhanced cellular migration properties [42, 43]. In that case, it was proven that the overexpression of these two transcription factors promoted the migration of MSCs.

The migration of cells through a vessel wall constitutes a distinct challenge. It was shown that adhesion molecules play an important role in this process [44–46]. It has been reported that viral transduction of ITGA-4 was sufficient to increase the homing of MSCs to bone marrow [47]. However, it is not clear whether this phenomenon could be accomplished when targeting the brain parenchyma, although there is encouraging data from* in vitro* studies [48].

Finally, there are examples of MSCs dual target engineering in order to enhance vessel wall migration. In that case, MSCs simultaneously modified with two mRNAs for PSGL-1 and SLeX were compelled to produce functional ligands for P-selectins and E-selectins, which altogether resulted in improved inflamed tissue homing, like inflamed ear [49] and spinal cord [50].

## 3. Modifications of MSCs to Combat Senescence

**Figure 3:**
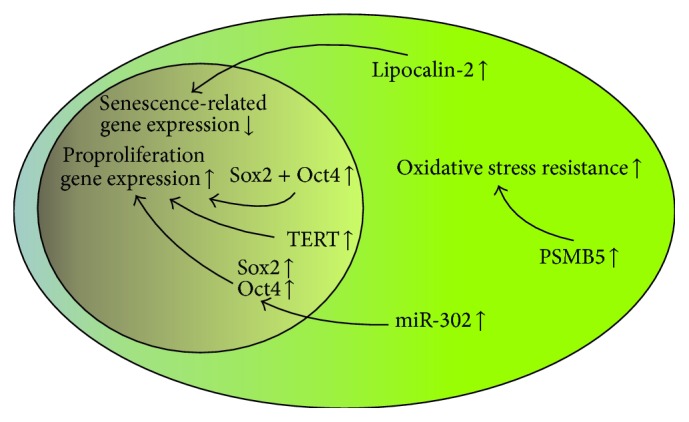
Proliferative and differentiation potential of MSC engineering. An improvement in the proliferative hallmarks of MSCs could be accomplished by the stimulation of proproliferation genes regulated by Sox2 and Oct4 transcription factors. Furthermore, senescence-related genes could be silenced (i.e., lipocalin-2 production), or oxidative stress resistance might be enhanced (i.e., PSMB5).

Two transcription factors, Sox2 and Oct4, are involved in maintenance of the pluripotency and self-renewal abilities of embryonic stem cells [51, 52]. Previously, both factors were used to reprogram adult somatic cells into induced pluripotent stem cells [53]. In addition, there are several reports that these two transcription factors were efficiently applied to engineer MSCs. Fan et al. found that bone marrow-derived MSCs simultaneously overexpressing Sox2 and Oct4 genes were characterized by improved proliferative and differentiation potential compared to control cells [54] (Figure 3). Similar beneficial effects related to Sox2 and Oct4 overexpression were reported for adipose-derived MSCs [55]. In that case, the transduced cells were more proliferative than controls, with increased differentiation abilities for adipocytes and osteoblasts. However, in a different study, bone marrow-derived MSCs were found to be efficiently engineered with the Sox2 gene, successfully retained in an undifferentiated state, but, in this case, the osteogenic and adipogenic differentiation potential of engineered cells was inhibited [56]. It has been shown that overexpression of the Oct4 gene in MSCs resulted in an increased expression of other stemness genes, such as Sox2 [57]. The overexpression of the Sox2 and Oct4 genes could also be achieved by concomitant treatment with leukemia inhibitory factor (LIF) and transfection with one of the stem cell-specific miRNAs, miR-302 [58]. What is more, miR-302 reportedly induced proliferation and inhibited oxidant-induced cell death in human adipose-derived MSCs [59].

Telomerase reverse transcriptase (TERT) gene transfection is yet another strategy to prevent senescence in cultured MSCs. TERT is an RNA-dependent DNA polymerase, which synthesizes and extends telomeric DNA, thus sustaining the immortal phenotype of stem cells [60]. It has been previously shown that MSCs lack TERT gene expression during* in vitro* expansion [61]; for this reason, the TERT gene engineering was converted into an interesting approach to reverse senescence in cultured MSCs. In addition, the differentiation potential of TERT-transfected MSCs toward osteogenic and neural lineages was improved compared to native MSCs [62].

In a different study, TERT immortalized MSCs had enhanced proliferative capabilities, and the cell-cycle-related gene expression factors were elevated, preventing the transfected MSCs from cell-cycle arrest [63]. Since the proteasomal pathway is important in the maintenance of cellular homeostasis and its dysfunction may lead to replicative senescence, transfection of MSCs with the *β*-subunit of the mammalian proteasome complex (PSMB5) also resulted in inhibition of cellular senescence [64, 65]. Yet, two other examples of preventing the cellular senescence of MSCs include transfection with small interfering RNAs against the glucocorticoid receptor gene [66] and lipocalin-2 gene overexpression, which protects the pluripotency of MSCs under hypoxic conditions [67].

Finally, the proliferative capabilities of MSCs could be improved by overexpressing growth factor genes. However, certain growth factors could severely impair the therapeutic properties of MSCs [68].

## 4. Engineering of MSCs to Improve Survival

**Figure 4:**
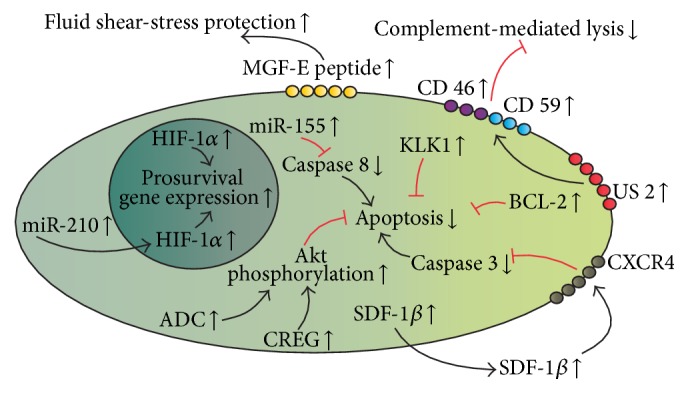
MSC prosurvival engineering. Prosurvival strategies primarily target the apoptosis process by downregulation of the elements involved in the apoptotic cascade (i.e., caspase 8 inhibition by miR-155). Other approaches involve the induction of prosurvival genes (i.e., HIF-1*α*), while still others ensure protection from fluid-stress or complement-mediated lysis.

Physiologically, MSCs migrate to the sites of injury, which are under hypoxic conditions, but, despite this physiological behavior, MSCs are also sensitive to the harsh local conditions encountered in the areas of their putative therapeutic action [69]. The survival of therapeutic cells is particularly important in injuries associated with hypoxia in the damaged tissue, such as myocardial infarction and stroke. For this reason, diverse prosurvival strategies have been developed to modify MSCs in order to prolong their survival in the target organ, giving them sufficient time to elicit beneficial effects (Figure 4).

Interestingly, SDF-1*β* was found to be a prosurvival player that enhanced cellular autophagy and decreased apoptosis in the SDF-1*β*-producing MSCs cultivated* in vitro* [70]. In addition, in a low oxygen concentration, a cardiac-type fatty acid binding protein was useful for the survival of the modified MSCs; however, the cell growth and proliferation of those cells were negatively affected [71].

Hypoxia-inducible factor-1*α* (HIF-1*α*) is a major regulator of the changes in the cellular metabolism caused by hypoxia [72]. HIF-1*α* regulates the activation of a broad range of genes involved in angiogenesis, erythropoiesis, cell proliferation, differentiation, and apoptosis in order to facilitate the adaptation of the cells to hypoxic conditions [73]. Therefore, the HIF-1*α* gene could be worth consideration as a target in the prosurvival approaches for MSC therapies, since beneficial results were seen in HIF-1*α*-engineered MSCs in trials with a mouse hind-limb ischemia model [74] and in a rat myocardial infarction model [75]. Furthermore, miRNA technology also has an application in this regard, since MSCs were modified to overexpress miR-210, which favored HIF-1*α* protein activity in the positive feedback regulatory loop that fosters the survival of the modified MSCs under hypoxic conditions [76, 77].

Apart from HIF-1*α*, other engineering solutions have been used to target selected proteins from defined signaling pathways, with a specific emphasis on apoptosis. There are several examples of MSC engineering approaches to produce proteins that could inhibit apoptotic signaling in the therapeutic cells by providing antiapoptotic signals mediated by Bcl-2 [78], a cellular repressor of E1A-stimulated genes (CREG) [79, 80], kallikrein (KLK1) [81, 82], angiotensin-converting enzyme 2 [83], arginine decarboxylase (ADC) [84], integrin-linked kinase (ILK) [85, 86], or protein kinase G1*α* [87]. The antiapoptotic effects could be obtained because of the silencing properties of small hairpin RNAs (shRNA) as well. For instance, the expression of the well-known proapoptotic factor caspase 8 gene was abolished after pre-miRNA-155-designed caspase 8 shRNA transfection into MSCs [88].

An attractive option that could be viewed as a group of prosurvival solutions is the protection of therapeutic MSCs from the negative influence of extravasated blood [89]. For example, the use of mechanogrowth factor E (MGF-E) peptide in the membranes of the MSCs might protect the transfected cells from improper fluid shear stress [90]. Another example is the data reporting that MSCs could be protected from the complement-mediated damage by overexpression of US2 protein from the human cytomegalovirus [91].

## 5. Future Perspectives

MSCs emerge as a very attractive cell type for clinical applications because of their availability in comparison to other cells and there are no elevated ethical problems associated with their harvesting. Additionally, these cells may be quite easily propagated in* in vitro* environments. The use of autologous MSCs would be one of the most convenient solutions, which would obviate many of the problems associated with the immunological aspects and adequate cell donor match.

However, in the case of autologous cells, a scant starting material might pose a serious problem. This issue could be addressed by the use of genetic engineering techniques aimed at the increase of the mitotic properties. In practice, this means avoiding senescence, which inevitably occurs during prolonged* in vitro* culture. Due to these techniques, a sufficient number of cells at the moment of transplantation could be achieved that, in turn, would pave the way to the enhanced therapeutic outcome.

In practice, the problem with an insufficient number for cells for autologous transplantation is caused by the fact that the need of cell therapy occurs mainly in the elderly. In these patients, the therapeutic potential and number of MSCs that might be obtained from biopsy is reduced, compared to young patients [92]. However, an appropriate stimulation can modulate these cells to convert them into suitable therapeutic material in spite of their old age. Once again, in the field of genetic engineering, methods could contribute to the increase in the cell number and enhancement of their therapeutic potential.

In addition, another group of problems is related to the proper targeting of therapeutic cells to ensure that the least amount of these valuable cells would be lost during the administration step and to ensure that as many of them as possible will be delivered to the site of action. It seems that one of the less traumatic ways of cell administration is an intravascular delivery route. In this case, securing MSCs from the negative influence of blood seems to be pivotal, followed by proper tissue targeting, in order to prevent additional cell loss due to lung and lymphoid organ entrapment.

Subsequently, since these cells have to be used to treat injured areas, wherein native cells are heavily damaged, and in a location that is full of destructive factors, therapeutic cell protection at this stage seems to be also very important. Therefore, it is extremely important to ensure the prolonged survival of these cells after administration, so that they could remain long enough at a target site to have more time to act.

Finally, it appears that for the effective practical cell therapy, particularly for autologous transplants, all of the above mentioned elements should be incorporated at the same time. This would imply a need to use advanced techniques [93], modifying MSCs with multiples genes, at the same time ensuring the effective expression of each of them, and perhaps in some cases providing some sort of sequential induction of introduced gene expression. This aim might be achieved by the employment of different genetic material loaded nanostructures that, depending on the nanostructure composition, would potentially release their contents into the cytoplasm with different speed [94, 95].

## 6. Conclusions

The aim of this review was to bring attention to the importance of promigratory and prosurvival aspects when considering MSC-based therapies. The successful therapeutic use of MSCs depends on efficient cell delivery and adequate survival of therapeutic cells, as well as the unhindered differentiation capacity of engineered MSCs. It seems that, in practice, there would be a need for the simultaneous implementation of a combination of the presented genetic engineering solutions presented here, in order to achieve truly therapeutic goals. The coupling of modified genes might be potentially tailored to the needs of specific patients and contribute to personalized medicine.

## References

[1] Nowakowski A., Andrzejewska A., Janowski M., Walczak P., Lukomska B. (2013). Genetic engineering of stem cells for enhanced therapy. *Acta Neurobiologiae Experimentalis*.

[2] Nowakowski A., Walczak P., Janowski M., Lukomska B. (2015). Genetic engineering of mesenchymal stem cells for regenerative medicine. *Stem Cells and Development*.

[3] Annabi B., Lee Y.-T., Turcotte S. (2003). Hypoxia promotes murine bone-marrow-derived stromal cell migration and tube formation. *STEM CELLS*.

[4] Lin Y.-T., Chern Y., Shen C.-K. J. (2011). Human mesenchymal stem cells prolong survival and ameliorate motor deficit through trophic support in Huntington's disease mouse models. *PLoS ONE*.

[5] Janowski M., Engels C., Gorelik M. (2014). Survival of neural progenitors allografted into the CNS of immunocompetent recipients is highly dependent on transplantation site. *Cell Transplantation*.

[6] Janowski M., Kuzma-Kozakiewicz M., Binder D. (2008). Neurotransplantation in mice: the concorde-like position ensures minimal cell leakage and widespread distribution of cells transplanted into the cisterna magna. *Neuroscience Letters*.

[7] Janowski M., Walczak P., Kropiwnicki T. (2014). Long-term MRI cell tracking after intraventricular delivery in a patient with global cerebral ischemia and prospects for magnetic navigation of stem cells within the CSF. *PLoS ONE*.

[8] Habisch H.-J., Janowski M., Binder D. (2007). Intrathecal application of neuroectodermally converted stem cells into a mouse model of ALS: limited intraparenchymal migration and survival narrows therapeutic effects. *Journal of Neural Transmission*.

[9] Janowski M., Lyczek A., Engels C. (2013). Cell size and velocity of injection are major determinants of the safety of intracarotid stem cell transplantation. *Journal of Cerebral Blood Flow and Metabolism*.

[10] Cui L.-L., Kerkelä E., Bakreen A. (2015). The cerebral embolism evoked by intra-arterial delivery of allogeneic bone marrow mesenchymal stem cells in rats is related to cell dose and infusion velocity. *Stem Cell Research and Therapy*.

[11] Bayo J., Fiore E., Aquino J. B. (2014). Increased migration of human mesenchymal stromal cells by autocrine motility factor (AMF) resulted in enhanced recruitment towards hepatocellular carcinoma. *PLoS ONE*.

[12] Kang S. K., Shin I. S., Ko M. S., Jo J. Y., Ra J. C. (2012). Journey of mesenchymal stem cells for homing: strategies to enhance efficacy and safety of stem cell therapy. *Stem Cells International*.

[13] Wagner W.,  Horn P., Castoldi M. (2008). Replicative senescence of mesenchymal stem cells: a continuous and organized process. *PLoS ONE*.

[14] Janowski M. (2009). Functional diversity of SDF-1 splicing variants. *Cell Adhesion and Migration*.

[15] Li Q., Zhang A., Tao C., Li X., Jin P. (2013). The role of SDF-1-CXCR4/CXCR7 axis in biological behaviors of adipose tissue-derived mesenchymal stem cells in vitro. *Biochemical and Biophysical Research Communications*.

[16] Janowski M., Lukomska B., Domanska-Janik K. (2011). Migratory capabilities of human umbilical cord blood-derived neural stem cells (HUCB-NSC) in vitro. *Acta Neurobiologiae Experimentalis*.

[17] Liu N., Tian J., Cheng J., Zhang J. (2013). Migration of CXCR4 gene-modified bone marrow-derived mesenchymal stem cells to the acute injured kidney. *Journal of Cellular Biochemistry*.

[18] Ma H.-C., Shi X.-L., Ren H.-Z., Yuan X.-W., Ding Y.-T. (2014). Targeted migration of mesenchymal stem cells modified with CXCR4 to acute failing liver improves liver regeneration. *World Journal of Gastroenterology*.

[19] Yang J.-X., Zhang N., Wang H.-W., Gao P., Yang Q.-P., Wen Q.-P. (2015). CXCR4 receptor overexpression in mesenchymal stem cells facilitates treatment of acute lung injury in rats. *The Journal of Biological Chemistry*.

[20] Yu J., Li M., Qu Z., Yan D., Li D., Ruan Q. (2010). SDF-1/CXCR4-mediated migration of transplanted bone marrow stromal cells toward areas of heart myocardial infarction through activation of PI3K/Akt. *Journal of Cardiovascular Pharmacology*.

[21] Wang Y., Deng Y., Zhou G.-Q. (2008). SDF-1*α*/CXCR4-mediated migration of systemically transplanted bone marrow stromal cells towards ischemic brain lesion in a rat model. *Brain Research*.

[22] Yu Q., Liu L., Lin J. (2015). SDF-1*α*/CXCR4 axis mediates the migration of mesenchymal stem cells to the hypoxic-ischemic brain lesion in a rat model. *Cell Journal*.

[23] Deng C., Qin A., Zhao W., Feng T., Shi C., Liu T. (2014). Up-regulation of CXCR4 in rat umbilical mesenchymal stem cells induced by serum from rat with acute liver failure promotes stem cells migration to injured liver tissue. *Molecular and Cellular Biochemistry*.

[24] Chen W., Li M., Cheng H. (2013). Overexpression of the mesenchymal stem cell Cxcr4 gene in irradiated mice increases the homing capacity of these cells. *Cell Biochemistry and Biophysics*.

[25] Marquez-Curtis L. A., Gul-Uludag H., Xu P., Chen J., Janowska-Wieczorek A. (2013). CXCR4 transfection of cord blood mesenchymal stromal cells with the use of cationic liposome enhances their migration toward stromal cell-derived factor-1. *Cytotherapy*.

[26] Won Y.-W., Patel A. N., Bull D. A. (2014). Cell surface engineering to enhance mesenchymal stem cell migration toward an SDF-1 gradient. *Biomaterials*.

[27] Cao Z., Zhang G., Wang F. (2013). Protective effects of mesenchymal stem cells with CXCR4 up-regulation in a rat renal transplantation model. *PLoS ONE*.

[28] Du Z., Wei C., Yan J. (2013). Mesenchymal stem cells overexpressing C-X-C chemokine receptor type 4 improve early liver regeneration of small-for-size liver grafts. *Liver Transplantation*.

[29] Liu N., Patzak A., Zhang J. (2013). CXCR4-overexpressing bone marrow-derived mesenchymal stem cells improve repair of acute kidney injury. *American Journal of Physiology—Renal Physiology*.

[30] Yang D., Sun S., Wang Z., Zhu P., Yang Z., Zhang B. (2013). Stromal cell-derived factor-1 receptor CXCR4-overexpressing bone marrow mesenchymal stem cells accelerate wound healing by migrating into skin injury areas. *Cellular Reprogramming*.

[31] Yu X., Chen D., Zhang Y. (2012). Overexpression of CXCR4 in mesenchymal stem cells promotes migration, neuroprotection and angiogenesis in a rat model of stroke. *Journal of the Neurological Sciences*.

[32] Nakamura Y., Ishikawa H., Kawai K., Tabata Y., Suzuki S. (2013). Enhanced wound healing by topical administration of mesenchymal stem cells transfected with stromal cell-derived factor-1. *Biomaterials*.

[33] Liu Y., Carson-Walter E., Walter K. A. (2014). Chemokine receptor CXCR7 is a functional receptor for CXCL12 in brain endothelial cells. *PLoS ONE*.

[34] Wang Y., Fu W., Zhang S. (2014). CXCR-7 receptor promotes SDF-1*α*-induced migration of bone marrow mesenchymal stem cells in the transient cerebral ischemia/reperfusion rat hippocampus. *Brain Research*.

[35] Li H., Jiang Y., Jiang X. (2014). CCR7 guides migration of mesenchymal stem cell to secondary lymphoid organs: a novel approach to separate GvHD from GvL effect. *Stem Cells*.

[36] Brat D. J., Bellail A. C., Van Meir E. G. (2005). The role of interleukin-8 and its receptors in gliomagenesis and tumoral angiogenesis. *Neuro-Oncology*.

[37] Kim S. M., Kim D.-S., Jeong C. H. (2011). CXC chemokine receptor 1 enhances the ability of human umbilical cord blood-derived mesenchymal stem cells to migrate toward gliomas. *Biochemical and Biophysical Research Communications*.

[38] Huang J., Zhang Z., Guo J. (2010). Genetic modification of mesenchymal stem cells overexpressing CCR1 increases cell viability, migration, engraftment, and capillary density in the injured myocardium. *Circulation Research*.

[39] Meng F., Rui Y., Xu L., Wan C., Jiang X., Li G. (2014). Aqp1 enhances migration of bone marrow mesenchymal stem cells through regulation of FAK and *β*-catenin. *Stem Cells and Development*.

[40] La Porta C. (2010). AQP1 is not only a water channel: it contributes to cell migration through Lin7/beta-catenin. *Cell Adhesion and Migration*.

[41] Maijenburg M. W., Gilissen C., Melief S. M. (2012). Nuclear receptors Nur77 and Nurr1 modulate mesenchymal stromal cell migration. *Stem Cells and Development*.

[42] Zhang X., Yan G., Diao Z., Sun H., Hu Y. (2012). NUR77 inhibits the expression of TIMP2 and increases the migration and invasion of HTR-8/SVneo cells induced by CYR61. *Placenta*.

[43] Yang S., Edman L. C., Sánchez-Alcañiz J. A. (2013). Cxcl12/Cxcr4 signaling controls the migration and process orientation of A9-A10 dopaminergic neurons. *Development*.

[44] Chamberlain G., Smith H., Rainger G. E., Middleton J. (2011). Mesenchymal stem cells exhibit firm adhesion, crawling, spreading and transmigration across aortic endothelial cells: effects of chemokines and shear. *PLoS ONE*.

[45] Matsushita T., Kibayashi T., Katayama T. (2011). Mesenchymal stem cells transmigrate across brain microvascular endothelial cell monolayers through transiently formed inter-endothelial gaps. *Neuroscience Letters*.

[46] Krstić J., Obradović H., Jauković A. (2015). Urokinase type plasminogen activator mediates Interleukin-17-induced peripheral blood mesenchymal stem cell motility and transendothelial migration. *Biochimica et Biophysica Acta - Molecular Cell Research*.

[47] Kumar S., Ponnazhagan S. (2007). Bone homing of mesenchymal stem cells by ectopic *α*4 integrin expression. *The FASEB Journal*.

[48] Feng Y., Yu H.-M., Shang D.-S., Fang W.-G., He Z.-Y., Chen Y.-H. (2014). The involvement of CXCL11 in bone marrow-derived mesenchymal stem cell migration through human brain microvascular endothelial cells. *Neurochemical Research*.

[49] Levy O., Zhao W., Mortensen L. J. (2013). mRNA-engineered mesenchymal stem cells for targeted delivery of interleukin-10 to sites of inflammation. *Blood*.

[50] Liao W., Pham V., Liu L. (2016). Mesenchymal stem cells engineered to express selectin ligands and IL-10 exert enhanced therapeutic efficacy in murine experimental autoimmune encephalomyelitis. *Biomaterials*.

[51] Jerabek S., Merino F., Schöler H. R., Cojocaru V. (2014). OCT4: dynamic DNA binding pioneers stem cell pluripotency. *Biochimica et Biophysica Acta (BBA)—Gene Regulatory Mechanisms*.

[52] Zhang S., Cui W. (2014). Sox2, a key factor in the regulation of pluripotency and neural differentiation. *World Journal of Stem Cells*.

[53] Takahashi K., Yamanaka S. (2006). Induction of pluripotent stem cells from mouse embryonic and adult fibroblast cultures by defined factors. *Cell*.

[54] Fan Y. X., Gu C. H., Zhang Y. L. (2013). Oct4 and Sox2 overexpression improves the proliferation and differentiation of bone mesenchymal stem cells in Xiaomeishan porcine. *Genetics and Molecular Research*.

[55] Han S.-M., Han S.-H., Coh Y.-R. (2014). Enhanced proliferation and differentiation of Oct4- And Sox2-overexpressing human adipose tissue mesenchymal stem cells. *Experimental and Molecular Medicine*.

[56] Schönitzer V., Wirtz R., Ulrich V. (2014). Sox2 is a potent inhibitor of osteogenic and adipogenic differentiation in human mesenchymal stem cells. *Cellular Reprogramming*.

[57] Palma C. S., Tannous M. A., Malta T. M., Russo E. M. S., Covas D. T., Picanço-Castro V. (2013). Forced expression of OCT4 influences the expression of pluripotent genes in human mesenchymal stem cells and fibroblasts. *Genetics and Molecular Research*.

[58] Taha M. F., Javeri A., Rohban S., Mowla S. J. (2014). Upregulation of pluripotency markers in adipose tissue-derived stem cells by miR-302 and leukemia inhibitory factor. *BioMed Research International*.

[59] Kim J. Y., Shin K. K., Lee A. L. (2014). MicroRNA-302 induces proliferation and inhibits oxidant-induced cell death in human adipose tissue-derived mesenchymal stem cells. *Cell Death and Disease*.

[60] Kong F., Zheng C., Xu D. (2014). Telomerase as a ‘stemness’ enzyme. *Science China Life Sciences*.

[61] Baxter M. A., Wynn R. F., Jowitt S. N., Wraith J. E., Fairbairn L. J., Bellantuono I. (2004). Study of telomere length reveals rapid aging of human marrow stromal cells following in vitro expansion. *Stem Cells*.

[62] Tsai C.-C., Chen C.-L., Liu H.-C. (2010). Overexpression of hTERT increases stem-like properties and decreases spontaneous differentiation in human mesenchymal stem cell lines. *Journal of Biomedical Science*.

[63] Lee H.-J., Choi J.-H., Jung J., Kim J. K., Lee S. S., Kim G. J. (2014). Changes in PTTG1 by human TERT gene expression modulate the self-renewal of placenta-derived mesenchymal stem cells. *Cell and Tissue Research*.

[64] Lu L., Song H.-F., Wei J.-L. (2014). Ameliorating replicative senescence of human bone marrow stromal cells by PSMB5 overexpression. *Biochemical and Biophysical Research Communications*.

[65] Chondrogianni N., Stratford F. L. L., Trougakos I. P., Friguet B., Rivett A. J., Gonos E. S. (2003). Central role of the proteasome in senescence and survival of human fibroblasts. Induction of a senescence-like phenotype upon its inhibition and resistance to stress upon its activation. *The Journal of Biological Chemistry*.

[66] Hong L., Wei N., Joshi V. (2012). Effects of glucocorticoid receptor small interfering RNA delivered using poly lactic-co-glycolic acid microparticles on proliferation and differentiation capabilities of human mesenchymal stromal cells. *Tissue Engineering—Part A*.

[67] Bahmani B., Roudkenar M. H., Halabian R., Jahanian-Najafabadi A., Amiri F., Jalili M. A. (2014). Lipocalin 2 decreases senescence of bone marrow-derived mesenchymal stem cells under sub-lethal doses of oxidative stress. *Cell Stress and Chaperones*.

[68] Fierro F. A., Kalomoiris S., Sondergaard C. S., Nolta J. A. (2011). Effects on proliferation and differentiation of multipotent bone marrow stromal cells engineered to express growth factors for combined cell and gene therapy. *STEM CELLS*.

[69] Brandl A., Meyer M., Bechmann V., Nerlich M., Angele P. (2011). Oxidative stress induces senescence in human mesenchymal stem cells. *Experimental Cell Research*.

[70] Herberg S., Shi X., Johnson M. H., Hamrick M. W., Isales C. M., Hill W. D. (2013). Stromal cell-derived factor-1*β* mediates cell survival through enhancing autophagy in bone marrow-derived mesenchymal stem cells. *PLoS ONE*.

[71] Wang S., Zhou Y., Andreyev O. (2014). Overexpression of FABP3 inhibits human bone marrow derived mesenchymal stem cell proliferation but enhances their survival in hypoxia. *Experimental Cell Research*.

[72] Majmundar A. J., Wong W. J., Simon M. C. (2010). Hypoxia-inducible factors and the response to hypoxic stress. *Molecular Cell*.

[73] Semenza G. L. (2001). Hypoxia-inducible factor 1: control of oxygen homeostasis in health and disease. *Pediatric Research*.

[74] HoWangYin K.-Y., Loinard C., Bakker W. (2014). HIF-prolyl hydroxylase 2 inhibition enhances the efficiency of mesenchymal stem cell-based therapies for the treatment of critical limb ischemia. *STEM CELLS*.

[75] Huang B., Qian J., Ma J. (2014). Myocardial transfection of hypoxia-inducible factor-1*α* and co-transplantation of mesenchymal stem cells enhance cardiac repair in rats with experimental myocardial infarction. *Stem Cell Research and Therapy*.

[76] Chang W., Lee C. Y., Park J.-H. (2013). Survival of hypoxic human mesenchymal stem cells is enhanced by a positive feedback loop involving mir-210 and hypoxia-inducible factor 1. *Journal of Veterinary Science*.

[77] Xu J., Huang Z., Lin L. (2014). miR-210 over-expression enhances mesenchymal stem cell survival in an oxidative stress environment through antioxidation and c-Met pathway activation. *Science China Life Sciences*.

[78] Fang Z., Yang Q., Luo W. (2013). Differentiation of GFP-Bcl-2-engineered mesenchymal stem cells towards a nucleus pulposus-like phenotype under hypoxia in vitro. *Biochemical and Biophysical Research Communications*.

[79] Deng J., Han Y., Yan C. (2010). Overexpressing cellular repressor of E1A-stimulated genes protects mesenchymal stem cells against hypoxia- and serum deprivation-induced apoptosis by activation of PI3K/Akt. *Apoptosis*.

[80] Peng C. F., Han Y. L., Jie-Deng (2011). Overexpression of cellular repressor of E1A-stimulated genes inhibits TNF-*α*-induced apoptosis via NF-*κ*B in mesenchymal stem cells. *Biochemical and Biophysical Research Communications*.

[81] Li Y., Raman I., Du Y. (2013). Kallikrein transduced mesenchymal stem cells protect against anti-GBM disease and lupus nephritis by ameliorating inflammation and oxidative stress. *PLoS ONE*.

[82] Gao L., Bledsoe G., Yin H., Shen B., Chao L., Chao J. (2013). Tissue kallikrein-modified mesenchymal stem cells provide enhanced protection against ischemic cardiac injury after myocardial infarction. *Circulation Journal*.

[83] Liu F., Gao F., Li Q., Liu Z. (2014). The functional study of human umbilical cord mesenchymal stem cells harbouring angiotensin-converting enzyme 2 in rat acute lung ischemia-reperfusion injury model. *Cell Biochemistry and Function*.

[84] Seo S. K., Yang W., Park Y. M., Lee W. T., Park K. A., Lee J. E. (2013). Overexpression of human arginine decarboxylase rescues human mesenchymal stem cells against H_2_O_2_ toxicity through cell survival protein activation. *Journal of Korean Medical Science*.

[85] Mao Q., Lin C.-X., Liang X.-L., Gao J.-S., Xu B. (2013). Mesenchymal stem cells overexpressing integrin-linked kinase attenuate cardiac fibroblast proliferation and collagen synthesis through paracrine actions. *Molecular Medicine Reports*.

[86] Mao Q., Lin C., Gao J. (2014). Mesenchymal stem cells overexpressing integrin-linked kinase attenuate left ventricular remodeling and improve cardiac function after myocardial infarction. *Molecular and Cellular Biochemistry*.

[87] Wang L., Pasha Z., Wang S. (2013). Protein kinase G1 *α* overexpression increases stem cell survival and cardiac function after myocardial infarction. *PLoS ONE*.

[88] Liang Y., Lin Q., Zhu J. (2014). The caspase-8 shRNA-modified mesenchymal stem cells improve the function of infarcted heart. *Molecular and Cellular Biochemistry*.

[89] Moll G., Jitschin R., Bahr Lena L. (2011). Mesenchymal stromal cells engage complement and complement receptor bearing innate effector cells to modulate immune responses. *PLoS ONE*.

[90] Lv Y., Hao X., Sha Y., Yang L. (2014). Pretreatment with mechano-growth factor E peptide protects bone marrow mesenchymal cells against damage by fluid shear stress. *Biotechnology Letters*.

[91] Soland M. A., Bego M., Colletti E. (2013). Mesenchymal stem cells engineered to inhibit complement-mediated damage. *PLoS ONE*.

[92] Yao B., Huang S., Gao D., Xie J., Liu N., Fu X. (2015). Age-associated changes in regenerative capabilities of mesenchymal stem cell: impact on chronic wounds repair. *International Wound Journal*.

[93] Goldberg M., Langer R., Jia X. (2007). Nanostructured materials for applications in drug delivery and tissue engineering. *Journal of Biomaterials Science, Polymer Edition*.

[94] Li J., Yoong S. L., Goh W. J. (2015). In vitro controlled release of cisplatin from gold-carbon nanobottles via cleavable linkages. *Journal of International Journal of Nanomedicine*.

[95] Shen Y., Qiao H., Fan Q., Zhang S., Tang T. (2015). Potentiated osteoinductivity via cotransfection with BMP-2 and VEGF genes in microencapsulated C2C12 Cells. *BioMed Research International*.

